# Structural Basis of the Association of HIV-1 Matrix Protein with DNA

**DOI:** 10.1371/journal.pone.0015675

**Published:** 2010-12-23

**Authors:** Mengli Cai, Ying Huang, Robert Craigie, G. Marius Clore

**Affiliations:** 1 Laboratory of Chemical Physics, National Institute of Diabetes and Digestive and Kidney Diseases, National Institutes of Health, Bethesda, Maryland, United States of America; 2 Laboratory of Molecular Biology, National Institute of Diabetes and Digestive and Kidney Diseases, National Institutes of Health, Bethesda, Maryland, United States of America; University of South Florida College of Medicine, United States of America

## Abstract

HIV-1 matrix (MA) is a multifunctional protein that is synthesized as a polyprotein that is cleaved by protease during viral maturation. MA contains a cluster of basic residues whose role is controversial. Proposed functions include membrane anchoring, facilitating viral assembly, and directing nuclear import of the viral DNA. Since MA has been reported to be a component of the preintegration complex (PIC), we have used NMR to probe its interaction with other PIC components. We show that MA interacts with DNA and this is likely sufficient to account for its association with the PIC.

## Introduction

The HIV-1 matrix (MA) protein [1] is synthesized as part of the Pr55 Gag polyprotein that is cleaved to mature products during viral maturation. As part of Gag, MA is involved in virion assembly. The MA cleavage product is a component of the mature virion and enters infected cells along with viral RNA and other viral proteins. After reverse transcription, the viral RNA is associated with a large nucleoprotein complex called the preintegration complex (PIC) that is derived from the core of the infecting virion [2]. Viral proteins reported to be associated with the PIC include integrase (IN), reverse transcriptase (RT), capsid (CA), Vpr, and MA.

Integrase within the PIC plays an essential role in integrating the viral DNA into the host genome, but the function of other proteins is less clearly understood; some may simply be left over from the infecting virion and play no further role in viral replication. The role of MA has been particularly controversial. Peptides spanning the highly basic amino acids 25–33 of MA function as a nuclear localization signal (NLS) when fused to a reporter protein and it has been proposed that this NLS facilitates nuclear import of the PIC [3], [4]. However, subsequent studies slowed this putative NLS in MA is not essential for HIV-1 replication in non-dividing cells [5], [6], [7] and therefore is not essential for nuclear import of the PIC. Nevertheless, the N-terminal basic region of MA does seem to be required for maximum infectivity. Mutation of the N-terminal basic region of MA impairs infectivity and decreases circularization of viral DNA [8]. Although circular viral DNA is a dead end product that is not on the integration pathway, the effect of these mutations indicates that MA is functionally associated with the PIC and can influence the viral replication pathway after reverse transcription. Regardless of the function of MA for the PIC, its presence within the complex implies interaction with other components of the complex. MA binds DNA and binding requires the N-terminal basic region[9]. A direct interaction of MA and DNA may therefore account for its retention within the PIC. In this paper we use solution NMR spectroscopy to probe the interactions between MA and DNA. Using chemical shift perturbation mapping we confirm that MA interacts with DNA and identify the DNA binding surface of MA. Using paramagnetic resonance enhancement (PRE) measurements, we demonstrate that the interaction between MA and DNA is non-specific. The relative of orientation of MA on the DNA in the complex was ascertained from residual dipolar coupling measurements, and this data, together with the chemical shift perturbation map, allowed us to generate a model of the complex.

## Results

### Characterization of DNA binding of MA

**Figure 1:**
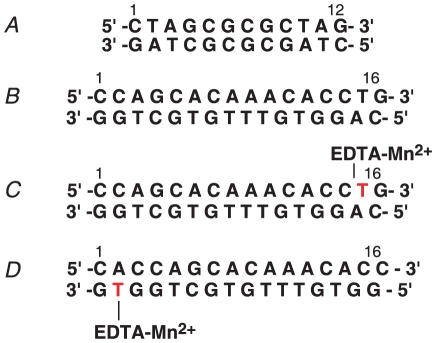
DNA oligonucleotide duplexes used in the current study. (A) and (B) are 12 mer and 16 mer DNA fragments used for chemical shift mapping and ITC measurements. DNA fragment (B) was also used for the RDC measurements. (C) and (D) are 16 mer DNA fragments containing dT-EDTA as indicated and were used for PRE measurements.

**Figure 2:**
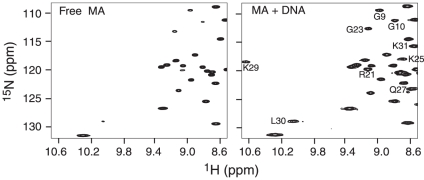
Interaction of MA with DNA monitored by ^1^H-^15^N HSQC spectroscopy. (A) Selected region of the ^1^H-^5^N-edited HSQC spectrum (recorded at a spectrometer frequency of 500 MHz) of free ^15^N-labeled MA (left panel) and ^15^N-labeled MA complexed with 16 mer DNA. Cross-peaks that are either shifted or intensified upon complexation are labeled.

**Figure 3:**
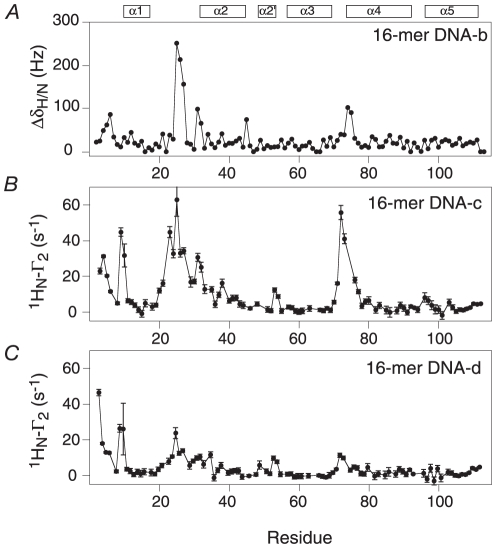
^1^H_N_/^15^N chemical shift perturbation mapping and PRE measurements on the interaction of ^15^N-labeled MA with DNA. (A) ^1^H_N_/^15^N chemical shift difference (Δδ_H/N_ = (ΔδN^2^+ΔδH_N_^2^)^1/2^] in Hz between the MA/DNA complex and free MA at a spectrometer frequency of 500 MHz. (B) and (C) ^1^H_N_-Γ_2_ PRE rates as a function of residue for ^15^N-labeled MA complexed to paramagnetically-labeled DNA; the oligonucleotides in (B) and (C) correspond to the oligonucleotides shown in Figs. 1C and D, respectively, with dT-EDTA-Mn^2+^ located at opposite ends of the DNA. The PRE data were recorded at a spectrometer frequency of 600 MHz.

Interactions between MA and DNA were studied by ^1^H_N_/^15^N chemical shift perturbation mapping, PRE measurements and isothermal titration calorimetry (ITC). A selected region of the 2D^ 1^H-^15^N heteronuclear single quantum coherence correlation (HSQC) spectrum for free MA and the same region for MA in complex with a 16 mer DNA (Fig. 1B) is shown in Fig. 2. The ^1^H_N_/^15^N chemical shift differences between MA in the DNA complex and free MA mainly involve a loop region between residues 22 and 32 (Fig. 3A). Under our solution conditions (25 mM potassium phosphate pH 6.5, and 50 mM NaCl) this loop exhibits chemical exchange in free MA as evidenced by line broadening of the ^1^H_N_/^15^N cross-peaks, but is fixed in one conformation upon DNA binding as evidenced by a narrowing of the corresponding ^1^H_N_/^15^N cross-peaks in the complex, suggesting that DNA binding stabilizes this region of MA. In addition, residues with substantial, albeit smaller, ^1^H_N_/^15^N chemical shift perturbations include the N-terminal tail, the N-terminal end of helix 2 and the N-terminal end of helix 4 (Fig. 3A). Exchange between free and DNA-bound MA is fast on the chemical shift scale, and the largest chemical shift difference observed between free and DNA-bound MA is ∼230 Hz (for the ^1^H_N_ resonance of Gly25). One can therefore conclude that the exchange rate between free and bound MA is ≥1500 s^−1^.

To determine the specificity of MA/DNA binding, ^1^H_N_/^15^N chemical shift perturbation of MA was compared with DNA oligonucleotides of different length and sequence. Titrations with the DNA duplexes shown in Figs. 1A and 1B exhibited similar ^1^H_N_/^15^N chemical shift perturbations (data not shown), indicating that MA binds DNA nonspecifically.

The nonspecific nature of binding of MA to DNA was further demonstrated by PRE experiments. The PRE is caused by magnetic dipolar interactions between a nucleus and the unpaired electron of a paramagnetic centre; this interaction results in an increase in the relaxation rate of the nuclear magnetization that is proportional to <*r* ^−6^>, where *r* is the distance between the nucleus of interest and the paramagnetic center [10]. The PRE effect is best measured as the transverse ^1^H_N_-Γ_2_ relaxation obtained by taking the difference in transverse ^1^H_N_-R_2_ relaxation rates in the paramagnetic and diamagnetic states [10], [11]. In this study, the paramagnetic centre was placed at either end of the DNA oligonucleotides as shown in Figs. 1C and 1D. The PRE profiles obtained with the paramagnetic centre at either end of the DNA oligonucleotides are similar (Figs. 3B and C), indicating that MA binds to multiple sites on the DNA in two orientations differing by 180° [12], [13]. The observation that the overall magnitude of the PREs observed with DNA-c are a factor of about two larger than those observed with DNA-d suggests that there is a small degree of sequence preference. In addition, the PRE profiles are broadly similar to the ^1^H_N_/^15^N chemical shift perturbation profile, with large PREs observed for the N-terminal tail, the loop between helices 1 and 2, and the N-terminal end of helix 3, providing independent confirmation of the binding interface identified by chemical shift mapping.

**Figure 4:**
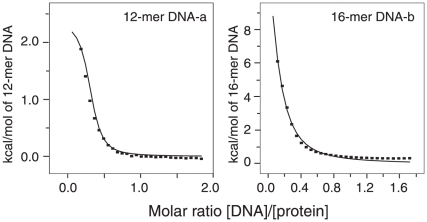
ITC measurements of the interaction of MA with DNA. 12 mer DNA (left panel) and 16 mer DNA (right panel) duplexes shown in Figs. 1A and B.

ITC experiments (Fig. 4) indicate that the 12 mer and 16 mer DNAs shown in Figs. 1A and B bind with approximately equal affinity, although the exact affinity could not be obtained as each DNA binds more than one MA.

### Model structure of MA/DNA complex

Residual dipolar couplings (RDC) measured for samples weakly aligned in dilute liquid crystalline media, such as phage pf1 [14], [15], are directly related to the orientation of bond vectors to the axes of the alignment tensor [16]. In the case of protein-DNA complexes aligned by negatively charged phage, it has been shown that alignment is dominated by the electrostatic properties of the DNA and that, providing the DNA is minimally distorted (i.e B-form and not significantly bent), the long axis of the DNA is approximately parallel to the principal axis of the alignment tensor[13]. This holds true even in the case of non-specific binding where the protein rapidly exchanges between all possible sites on the DNA [13].

**Figure 5:**
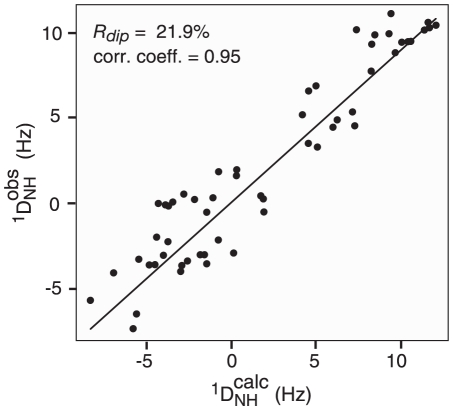
Comparison of observed (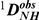) RDCs measured for the MA/DNA complex with those calculated (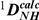) from the crystal structure of free MA. The dipolar coupling R-factor (defined as the ratio of the rms deviation between observed and calculated values and the expected rms deviation if the vectors were randomly distributed given by 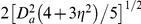 [28], where *D_a_* is the magnitude of the principal component of the alignment tensor and η the rhombicity) is 21.9%. The X-ray coordinates were taken from [17] (PDB code 1HIW), and addition of backbone amide protons and best-fitting of RDCs by singular value decomposition (SVD) was carried out using Xplor-NIH [18]. The values of 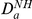 and η are 5.6 Hz and 0.3, respectively. The RDC data were measured using 11 mg/ml phage pf1 at a spectrometer frequency of 800 MHz, and a large excess of DNA was employed to ensure that all MA present was bound to DNA and only one MA molecule was bound per DNA duplex.

^1^D_NH_ backbone RDCs were measured for 66 well resolved residues of MA in the DNA complex, extending from residues 9 to 110. The sample used for RDC measurements contained 0.3 mM MA plus 2 mM 16 mer DNA, and the large excess of DNA compared to MA ensured that all MA was bound to DNA and that only one MA was bound per DNA duplex. Residues with overlapping or partially overlapping cross-peaks in the ^1^H-^15^N HSQC spectrum and highly mobile residues at the N and C-termini were not considered for the analysis. The measured RDCs were fitted against the 2.3 Å resolution crystal structure of MA (PDB code 1HIW)[17] by singular value decomposition (SVD) using Xplor-NIH [18], and a comparison of observed and calculated RDCs is shown in Fig. 5. The dipolar coupling R-factor is 21.9%, which is what one would expect for a crystal structure in the 2–2.5 Å resolution range [19], [20], indicating that the structure of MA in the MA/DNA complex is very similar to that found in the crystal structure (with the exception that the C-terminal end of MA from residue 110 onwards is disordered in solution but adopts a helical conformation in the crystal due to crystal packing forces). The magnitude of the principal component of the alignment tensor, D_a_^NH^, is 5.6 Hz and the rhombicity is 0.3. The low value of the rhombicity indicates that alignment of the MA/DNA complex is close to axially symmetric as expected if alignment is dominated by the electrostatic properties of B-form, essentially straight DNA [13]. [It should be noted that the SVD fit of the RDCs to the coordinates of the NMR structure (PDB code 2HMX[21]) determined from conventional nuclear Overhauser enhancement measurements results in rather poor agreement with a dipolar coupling R-factor of 61%, indicating that the accuracy of the NMR coordinates is rather low).

**Figure 6:**
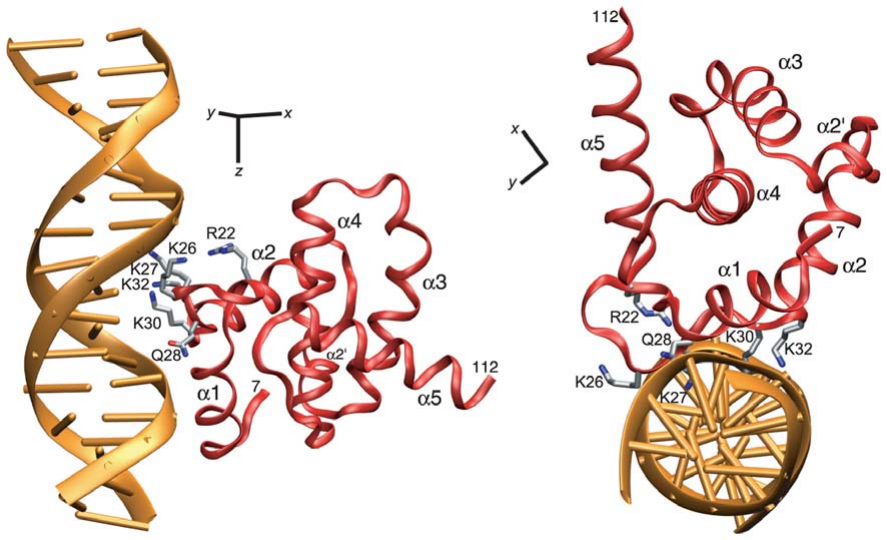
Model of the non-specific MA/DNA complex built on the basis of^ 1^H_N_/^15^N chemical shift perturbation, PRE and RDC measurements. The chemical shift perturbation and PRE measurements delineate the DNA binding surface on MA, while the RDC data permit one to align MA relative to the long axis of the DNA. Alignment of protein/DNA complexes where the DNA is essentially undistorted B-form DNA is dominated by the electrostatic properties of the DNA and the principal (*z*) axis of the alignment tensor is known to be essentially parallel to the long axis of the DNA [13]. The DNA is shown as a gold ribbon, MA as a red backbone tube with selected side chains as sticks, and the axes of the alignment tensor in black.

Knowing the regions of MA that are in close proximity to the DNA from both the ^1^H_N_/^15^N chemical shift perturbation map (Fig. 3A) and the PRE profiles (Figs. 3B and C), together with knowledge that the principal axis (i.e. the *z* axis) of the alignment tensor is approximately parallel to the long axis of B-form DNA, enables one to orient the protein on the DNA and obtain a crude model of the MA/DNA complex (Fig. 6). As the loop residues between residues 22 and 32 exhibit the largest ^1^H_N_/^15^N chemical shift perturbations upon DNA binding, we orientated the protein so that these loop residues face the DNA major groove while keeping the *z* axis of the alignment tensor along the long axis of the DNA. The N-terminal end of helix 2 also contacts the major groove, the N-terminal ends of helices 1 and 4 are close to the phosphate backbone, and the N-terminal tail is close to the minor groove, consistent with the chemical shift perturbation and PRE data shown in Fig. 3. As shown in Fig. 6, positively charged residues, including R22, K27, K27, Q28, K30 and K32 have reasonable contacts with the DNA major groove.

## Discussion

The basic N-terminal region of MA modulates multiple steps in HIV-1 replication and is required for maximal infectivity. It is not surprising that this positively charged surface can play roles in binding to RNA, DNA, and membranes, making the phenotypes of mutations in this region difficult to interpret. The interaction of MA with DNA is stable at least up to 200 mM NaCl and is therefore expected to occur under physiological conditions; indeed we found it necessary to include 500 mM NaCl in buffers during the early stages of purification to prevent co-purification with DNA. Although a function for MA as a predominant determinant of nuclear import is not supported by the preponderance of evidence, MA is a component of the HIV-1 PIC and can influence the fate of the viral DNA [8]. The interaction of MA reported here is likely sufficient to account for the retention of MA in the PIC even in the absence of an interaction with other protein components.

## Materials and Methods

### Protein Expression and Purification

HIV-1 p17 (MA) was cloned into a modified pET-32a vector [22] to express a thioredoxin fusion protein with a His_6_ tag in *E.coli* BL21(DE3) (Novagen, La Jolla, CA). The construct was verified by DNA sequencing. *E. coli* transformed with the plasmid were grown in minimal medium (with ^15^NH_4_Cl and ^13^C_6_-glucose as the sole nitrogen and carbon sources, respectively), induced with 1 mM isopropyl D-thiogalactopyranoside (IPTG) at A_600_ = 1.0, and harvested by centrifugation 3 h after induction. After harvesting, the cell pellet was resuspended in 50 ml (per liter of culture) of 50 mM Tris, pH 7.4, 500 mM NaCl, 10 mM imidazole, and 1 mM phenylmethylsulfonyl fluoride. The suspension was lysed by two passages through a microfluidizer and centrifuged at 10,000×g for 40 min. The supernatant fraction was loaded onto a HisTrap HP column (5 ml; GE Healthcare), and the fusion protein was eluted with a 100 ml gradient of imidazole (25–500 mM). The elution buffer also contained 500 mM NaCl. At lower NaCl concentrations (200 mM) the eluted protein was contaminated with DNA molecules, but maintaining 500 mM during washing and elution overcame this problem. The fusion protein was then dialyzed against 20 mM Tris, pH 8.0, and 200 mM NaCl, and digested with thrombin (10 NIH units/mg of protein). Thrombin was removed by passage over a benzamidine sepharose column (GE Healthcare). The cleaved His_6_-thioredoxin was removed by loading the digested proteins over a HisTrap HP column. MA was further purified by gel filtration on a Sephadex-75 gel filtration column (GE Healthcare) equilibrated with 25 mM potassium phosphate, pH 6.5, and 50 mM NaCl. For ITC studies, MA was eluted from a Sephadex-75 gel filtration column equilibrated with 50 mM Tris pH 7.5, 50 mM NaCl and 2 mM 2-mercaptoethanol. The buffer used for all NMR samples in this report was 25 mM potassium phosphate, pH 6.5, 50 mM NaCl, and 2 mM 2-mercaptoethanol in 95% H_2_O/5% D_2_O.

### NMR Spectroscopy

NMR spectra were recorded at 27°C on Bruker DMX500, DRX600 and DRX800 spectrometers. Spectra were processed using the program NMRPipe [23], and analyzed using the programs PIPP, CAPP, and STAPP [24]. Backbone ^1^H, ^15^N, and ^13^C resonances of free MA, and MA complexed to DNA was achieved by means of through-bond heteronuclear scalar correlations along the protein backbone using three-dimensional HN(CO)CACB and HNCACB experiments. Samples for backbone resonance assignments comprised either 0.5 mM U-[^13^C/^15^N]-MA or 0.5 mM U-[^13^C/^15^N]-MA plus 2 mM 16 mer DNA as shown in Fig. 1B. The interaction between MA and DNA was monitored by monitoring the changes in ^1^H_N_/^15^N cross-peaks in the ^1^H-^15^N HSQC spectra of U-[^15^N]-MA upon titration of unlabeled DNA. To determine the specificity of the interaction, two DNA oligonucleotides of different length and sequence were used for titration. The specificities were also analyzed using PRE measurements (see below).

Samples for PRE studies comprised 0.15 mM MA and 0.3 mM duplex DNA with metal chelated EDTA-derivatized deoxythymidine (dT-EDTA) near either the 5′ or 3′ ends of the DNA (Fig. 1), in 25 mM potassium phosphate, pH 6.5 and 50 mM NaCl. Synthetic oligonucleotides were purchased from Midland Certified Reagent and were purified as described previously [12], [25], [26]. ^1^H_N_ transverse PRE rates (Γ_2_) were obtained by taking the difference in ^1^H_N_-R_2_ transverse relaxation rates between paramagnetic (dT-EDTA conjugated to Mn^2+^) and diamagnetic (dT-EDTA conjugated to Ca^2+^) samples using a 2D ^1^H-^15^N HSQC-based experiment as described previously [11].

Residual dipolar couplings (RDC) of MA in complex with DNA were obtained from samples aligned in a liquid crystal medium of filamentous phage (12 mg/ml) [14], [15], by taking the difference in the one-bond N-H splitting between aligned (pf1) and isotropic (water) samples measured using 2D in-phase/anti-phase ^1^H-^15^N HSQC experiments [27] at a ^1^H frequency of 800 MHz. The sample used for RDC measurements contained 0.3 mM MA plus 2 mM 16 mer DNA. The large excess of DNA compared to MA ensures that all MA is bound to DNA and that only one MA is bound per DNA duplex.

### Isothermal titration calorimetry

Isothermal titration calorimetry (ITC) was performed using a VP-ITC calorimeter (Microcal, Inc.). All samples for ITC experiments were pre-equilibrated in 50 mM Tris, pH 7.5 containing 50 mM NaCl and 2 mM 2-mercaptoethanol, and 0.5 mM DNA (12 mer or 16 mer, Fig. 1) and titrated with 6 mM DNA in the syringe at 27°C. Analysis of the data was performed using the Origin™ software provided with the instrument.
